# Pathology and Classification of Pulpless Teeth, without Regard to Abscessed Conditions

**Published:** 1899-09

**Authors:** A. H. Peck

**Affiliations:** Chicago


### THE DENTAL REGISTER.

VOL. LIII.] SEPTEMBER, 1899. [No. 9.

### COMMUNICATIONS.

### Pathology and Classification of Pulpless Teeth, Without Regard to Abscessed Conditions.

BY A. H. PECK, M.D., D.D.S., CHICAGO.

It is my purpose in this paper to present the various pathological conditions leading up to the death of the pulp, and to discuss the meaning of various expressions used in considering cases of pulpless teeth; also, to consider them in classes as based upon the various pathological conditions present in different cases; also, to consider the principles of their treatment, or the various therapeutic actions of drugs as made necessary by the different pathological conditions that may be present.

The first pathological condition leading up to the death of a pulp is *irritation*. Irritation can only be transmitted to the pulp itself through the medium of one channel, and that is the "fibrils of Tomes," except in those cases where caries extends to and exposes the pulp to direct contact with irritating substances from without, in which cases there has been no previous manifestation of irritation, and which class of cases is of rare occurrence.

The walls of the pulp chamber are lined throughout their entire surfaces with a layer of columnar cells, called odontoblasts. From the odontoblasts extend fibrils, organic material, in vital structure like the cells themselves, which are called the "fibrils of Tomes," as above referred to. These fibrils occupy space throughout the central portions of the dentinal tubules. I may, perhaps, best illustrate this point by referring to the nature and peculiar characteristics of the cells called *amoeba*. The amœboid cells are the lowest form of animal life that we know anything about. While they do not possess the attributes and characteristics in a large measure of regularly organized animal life, some of them they do possess, and one is their sensitivity. These cells are very sensitive to any changes taking place about them. You place them on a glass and observe them under the microscope, and it will be seen that any little jar to the table irritates them, and they immediately draw themselves up into little balls and thus remain motionless, if not further disturbed, until seemingly the danger has passed, when they will begin to move again. These cells are merely minute particles of vital matter, of protoplasm. So are the fibrils of Tomes composed only of vital matter. As the amoeboid cells are sensitive, so are the cells of the human system sensitive. They seem to be constantly on the watch for any interference with the performance of their normal functions; the disturbance irritates them, and this irritation is thus transmitted to the network system of nerves throughout the body, and in turn by the nerve system to the sensorium. It is the same with the dentine tissue. When you are cutting the dentine you are cutting the fibrils of Tomes, and the irritation thus imparted is transmitted by them to the odontoblasts (which are in close physiological relation with the pulp proper), and by the odontoblasts to the pulp, and by the pulp to the sensorium. In this way the dentine is sensitive—not sensitive in the physical sense or in the literal sense of the word, because there are no nerves in it, but it is sensitive in the physiological sense of the term.

Following irritation of the pulp, hyperaemia develops. In many cases the pulps die while in this pathologic state, by infarction—the cutting off of the circulation by an embolus. If death does not occur in this way, true inflammation follows, during which process the pulp undergoes all the pathological changes and conditions manifested generally in parts while passing through the inflammatory state, with the one exception that it does not swell; it is encased in bony walls and can not swell. In inflammation the normal structural characteristics of the tissue are almost completely lost, so great are the changes that occur. Some portions of the blood-vessels are widely distended, while other portions are much contracted; the areolar tissue is filled with the inflammatory products; the cells change form, their spindle-like projections disappear and they become rounded, after which their number is largely augmented by subdivision; the circulation is cut off and the pulp dies. The dead tissue may remain thus indefinitely without causing trouble; not until it is infected with micro-organisms will putrefaction occur. This accounts for the fact that some teeth containing dead pulps remain for years without causing trouble. No doubt, in the majority of cases, the micro-organisms gain direct access to the pulp chamber through external openings such as carious cavities; when not in this way, they are carried by the circulation of the blood and enter the pulp chamber through the opening in the apex of the root. Do not form the idea that micro-organisms are always present in human blood, for this is not the case. Indeed, at the present time it is the teaching of pathologists generally that micro-organisms are not present in normal human blood except by accident.

If by any abrasion of the surface of the skin they are permitted to enter the circulation, and, while traversing the system through this medium are not brought in contact with an inflamed area at any point, or with an otherwise favorable field for their development, they are destroyed. They can not retain their virulency for any length of time, being carried about in the blood current.

When the proper class of micro-organisms is brought in contact with the dead pulp tissue, under favorable conditions, the process of putrefaction is inaugurated; sulphuretted hydrogen gases, chiefly, are generated, and the entire mass of dead tissue is transformed into what is properly termed infectious material, which simply means, in this connection, material that contains the necessary elements to infect, or to cause to become diseased, tissue otherwise healthy. How frequently we hear this tissue in this condition referred to as "septic" material. Many use the two terms, "septic" and "infectious," as having like meaning. In the early times, comparatively speaking, before any definite knowledge of micro-organisms, all these pathological conditions of dead matter were referred to as septic. However, after the dissemination of knowledge regarding micro-organisms and their influence in producing disease, the word "infectious" came into general use for reference to dead matter that had been invaded by micro-organisms, or, in other words, that possessed the necessary element to enable it to transmit disease to otherwise healthy tissue. The term "septic" was thenceforth used in a restricted sense. Permit me to illustrate: A felon occurs on one's finger, or a boil any place on your hand or arm; pus micro-organisms are present and pus formation takes place. In order for another tissue to be infected by this pathological condition, it is necessary that a portion of the liquid—the pus—or the micro-organisms themselves shall be brought in contact with it. But there are other elements being continually formed—ptomaines, waste products which within themselves contain no micro-organisms. The human economy is constantly endeavoring to rid the system of these elements. They are carried away from the suppurative process through the channels of the lymphatic system of vessels. The lymphatic glands at the elbow, or more especially at the axilla, become inflamed, sore and swollen as a result of the poisonous waste material passing through them. This material, however, does not possess living germs, so that suppuration will not occur in the glands unless the infecting element—micro-organisms—is brought in contact with them through some other channel. This is "septic" material, and thus, in this restricted sense, should the term "septic" be used at the present time.

So many seem also to have a misconception of, or, perhaps better said, are careless in the proper use of the terms "antiseptic," "disinfectant," and "germicide," that I trust it will not be considered out of place if we give this brief attention. This phase of the subject also carries us back to prehistoric times, so far as micro-organisms are concerned. Up to the time that pathogenic micro-organisms and their connection with disease was understood, the term "germicide" was not in use. "Antiseptic" and "disinfectant" were the only expressions in use in this connection. The word "antiseptic" was used with reference only to those medicinal agents which prevented the decay, or the decomposition or putrefaction of the various substances. The word "disinfectant" was used to designate those medicinal agents which would not only prevent the further decomposition of these materials and the formation of gases and mephitic odors, but which would also destroy the noxious properties of fermentation and putrefaction. After a knowledge of the existence of pathogenic micro-organisms was had, and their relation to the history of the causation of disease became familiar, the word "germicide" came into use. This term was used to designate medicinal agents that would not only prevent the decomposition and putrefaction of substances through the agency of micro-organisms, but that would destroy the potency, the life, of the germs themselves. In substantially these meanings should these terms be used at the present time.

*Antiseptic*, as applied to medicinal agents, should be used only in the sense of preventing the development of micro-organisms—preventing putrefaction of nitrogenous substances by holding in check the development of germs. It will be readily understood that all germicides are antiseptic, but that all antiseptics are by no means necessarily germicides.

First, let us consider the class of pulpless teeth which seems most favorable for treatment and immediate root-canal filling. Occasionally teeth will present, the pulps of which have died and in the canals of which no remains of tissue or debris are found. Through some process executed by the mysteries of the human economy, the tissue has entirely disappeared. This condition may occur in teeth in the crowns of which no cavities exist. In these cases the pulps have become irritated from other causes than the poisonous products of carious cavities. They may have suffered shock from a blow more or less severe, or may have been subjected to extreme thermal changes; a low form of irritation is thus inaugurated, and continues until, eventually, the condition of hyperaemia develops and the pulp dies from infarction, without the presence of the inflammatory process.

In the root-canals of these teeth no moisture or odor is found; the walls are dry and clean, seemingly in an aseptic condition. It is the common teaching in these cases, and in the main is no doubt correct, that the apices of the roots have become encysted; a thickened membrane has formed over them, sealing tightly the foraminal openings, thus preventing moisture entering the root-canals from the apical spaces. Comparatively, these cases are not numerous. During my practice I have observed but four of them. It is the teaching of some to make immediate root-canal fillings in all cases belonging to this class of pulpless teeth. I have followed this practice in a few cases and have had no trouble. I think, however, it is better practice to treat with some non-irritating antiseptic a few days previous to filling. One is not positive the canals are aseptic, even though they contain no debris, moisture or odor. A few germs may be lurking about awaiting a favorable opportunity to cause trouble. Remember the old adage, and one more apropos was never uttered—"An ounce of prevention is worth a pound of cure."

The use of the stronger agents, as to antiseptic power, is not necessary in these cases; only the milder ones, and those, too, which are known to be non-irritating to soft tissues, so the latter, in the apical spaces, will not be irritated as the agents are absorbed by them.

Another class of pulpless teeth that presents for treatment is comprised of those in which the pulp tissue remains in the root-canals, and which, apparently, has not been infected with pathogenic micro-organisms. There is no indication of putrefaction such as the generation of odors, and the tissues in the apical spaces are not irritated. It is also always best to exercise due caution in the treatment of these cases. There is the same uncertainty as to the actual condition of the contents of the canals of these teeth as attaches to those of the first class. One is not positive that no pathogenic micro-organisms are present, and in most cases will find it better practice to fall a little short of a complete removal of the debris at the first sitting. A non-irritating antiseptic should be placed in the canal for a few days, after which thorough cleansing may be effected. By proceeding in this way the chances of disturbing the tissues in the apical spaces, resulting in future trouble, are reduced to the minimum. I would not say it is impossible to cleanse and make immediate fillings in the root-canals of such teeth, with no trouble resulting. No doubt we have all made immediate operations in these cases, and have been successful. However, because of the uncertainty of the conditions, I prefer in most cases to treat a few days previous to filling.

Another class of pulpless teeth that presents for treatment comprises those, the tissue in the root-canals of which has been infected with pathogenic micro-organisms. The process of decomposition has been inaugurated and odors are being generated. There is as yet, however, no irritation of the tissues in the apical spaces. This is a class of cases in which no operator, in my judgment, is justified in attempting a complete removal of the contents of the root-canals at the first sitting, to say nothing of the advisability of making immediate root-canal fillings. No matter how expert one may be in the use of broaches, or how careful to prosecute the cleansing process under antiseptic precautions, the possibility, and in most cases the probability, exists of forcing a quantity, however minute, of the infectious material through the foraminal openings, and thus infecting the tissues in the apical spaces. A complete removal of the contents of the root-canals of these teeth should not be attempted at the first sitting, and the broaches should be carefully used, with a firm grasp, to prevent them from slipping and forcing the infectious material through the foraminal openings. After the debris has been removed for two-thirds or three-fourths the distance to the apices of the roots, the case should be treated for a few days with a non-irritating agent that is known to be a potent germicide as well as an antiseptic. After this the remaining debris may be removed with little or no danger of irritating the apical tissues.

Another class of pulpless teeth, and the last one I will consider, comprises all cases the pulp tissue of which has been infected with pathogenic micro-organisms and the tissue is in the advanced stages of the putrefactive process. Mephitic odors are present to a degree which is decidedly unpleasant. The apical tissues have been infected and are inflamed; the inflammation has extended to other portions of the peridental membrane, and the case presents a pericementitis which is general.

In these cases one should not hesitate to completely remove, by mechanical and medicinal means, the contents of the root-canals. This should be done carefully but thoroughly. If a portion of the infectious material should be forced through the foramen, no special harm is likely to result, in most cases, for the apical tissues are already infected. However, one should exercise judgment in this connection, also due care, because abscesses have been known to develop in a surprisingly short time after the cleansing process has been commenced in these cases. All the elements necessary to pus formation are probably present, and, when disturbed, the process of fermentation is inaugurated and an abscess is the result. This probable termination makes it necessary that extreme care be exercised in removing the contents of the root-canals.

The medicines used in these cases should not only be potent antiseptics, but should be germicides as well. Micro-organisms are known to be present, and it is not only necessary that they be restrained from developing, but that they be destroyed. These agents should not only be non-irritating to soft tissue, but should be those whose therapeutic action is that of sedatives. The apical tissues are inflamed, and if irritating agents are used they serve to further aggravate these pathological conditions. If sedative agents are used, they will impart to the inflamed apical tissues a soothing influence; will serve to reduce the inflammation, and thus assist materially in one's efforts to restore the tissues to their normal condition. Agents for the treatment of these cases should also possess the properties of deodorants. In cases of long standing the dentinal tubules will be saturated with mephitic odors and gases, and it is necessary that these be destroyed.

In most cases of this class local external treatment is desirable and even necessary. In addition to the peridental membrane, the gum tissue is inflamed and swollen. The patient may be suffering with constant pain; at any rate, severe pain is occasioned by the slightest pressure upon the tooth. Under these conditions counter-irritation is always indicated. The free local use of appropriate antiphlogistics should not be neglected. Astringents also are of value in assisting to reduce the swelling.

Frequently systemic treatment in a moderate degree is desirable and productive of good results. In this connection, the chief indications in these cases are to slow the blood current, to quiet the nervous system, and to stimulate the secretions and excretions. This may be done by the use of tincture of aconite and veratrum viride, minim doses every half hour, and tincture of gelsemium, one-half minim doses every hour till six minims have been taken. Antikamnia, a proprietary mixture which acts on all parts of the system as a sedative, given in five-grain tablets every half hour until five or six of the tablets have been taken, usually results in much benefit. Sulphide of calcium is generally a useful agent in this connection, in doses of one-tenth to one-half grain. If the patient is constipated, a laxative is indicated—a saline cathartic—and this should never be overlooked. A hot foot-bath just before retiring is helpful, and if the patient is still suffering pain and is especially nervous, nothing is better than the action of Dover's powder on retiring.

The above form of systemic treatment is of special value in cases of threatened formation of abscess, and especially in cases which have advanced to a stage where it seems impossible to prevent pus formation. By this treatment the patient will be greatly relieved, in most cases, from the pain he would otherwise suffer, even though pus continues to form.

